# High-mobility group protein box1 expression correlates with peritumoral macrophage infiltration and unfavorable prognosis in patients with hepatocellular carcinoma and cirrhosis

**DOI:** 10.1186/s12885-016-2883-z

**Published:** 2016-11-11

**Authors:** Qiang-Bo Zhang, Qing-an Jia, Hong Wang, Chun-Xiao Hu, Dong Sun, Run-De Jiang, Zong-Li Zhang

**Affiliations:** 1 Department of General Surgery, Qilu Hospital, Shandong University, 107 Wenhua West Road, Jinan, 250012 China; 2 Cancer Center, Institutes of Biomedical Sciences, Fudan University, Shanghai, 200032 China; 3 Department of Anesthesiology, Yidu Central Hospital, Weifang Medical University, Qingzhou, 262500 China

**Keywords:** High-mobility group protein box1, Tumor-associated macrophage, Hepatocellular carcinoma, Liver cirrhosis, Prognosis

## Abstract

**Background:**

High-mobility group protein box1 (HMGB1) is a pivotal factor in the development and progression of many types of tumor. Its role in hepatocellular carcinoma (HCC), and especially its correlation with intratumoral and peritumoral macrophage infiltration, remains obscure. We analyzed the potential roles and prognostic value of HMGB1 and explored the correlation between HMGB1 and macrophage infiltration in HCC using clinical samples.

**Methods:**

We reviewed clinicopathological and follow-up data on a cohort of 149 patients with HCC complicated with Hepatitis B-related cirrhosis. We measured the expression of HMGB1 and CD68 in tumoral and peritumoral liver tissues after curative resection and assessed the impacts of the tumor-associated macrophage (TAM) count and HMGB1 expression on clinicopathologic characteristics, overall survival (OS), and recurrence-free survival (RFS).

**Results:**

Ninety-four of the patients had elevated tumoral HMGB1 expression and 59 of the patients had elevated peritumoral HMGB1 expression, compared to only 4 patients with elevated peritumoral HMGB1 expression in 36 pateints with Hepatitis B virus (HBV)-negative HCC without liver cirrhosis (*p* < 0.001). The peritumoral HMGB1 expression levels were correlated with tumor invasiveness, BCLC stage, and recurrence. The degree of TAM infiltration was higher in peritumoral tissues with high HMGB1 expression than in peritumoral tissues with low HMGB1 expression (*p* < 0.001). There was no significant difference in TAM infiltration between tumoral tissues with high and low HMGB1 expression. Kaplan-Meier analysis showed that intratumoral HMGB1 overexpression was associated with poor OS, but not with RFS. High peritumoral HMGB1expression and TAM count, which correlated positively with tumor size and BCLC stage, were independent prognostic factors for OS (*p* < 0.001 and *p* = 0.017, respectively) and RFS (*p* = 0.002 and *p* = 0.024, respectively). Multivariate analyses indicated peritumoral HMGB1 expression (*p* = 0.014) and TAM count (*p* = 0.037), as well as tumor differentiation (*p* = 0.026), to be independent significant prognostic factors for RFS.

**Conclusions:**

High HMGB1 expression in peritumoral liver tissues correlated with peritumoral macrophage infiltration and had prognostic value in HCC, suggesting that peritumoral HMGB1 might show promise as a new biomarker to predict HCC progression.

**Electronic supplementary material:**

The online version of this article (doi:10.1186/s12885-016-2883-z) contains supplementary material, which is available to authorized users.

## Background

Hepatocellular carcinoma (HCC), the fifth most common cancer worldwide, ranks as the third leading cause of cancer death globally [1, 2]. Although surgical resection and liver transplantation are the main modalities of curative treatment to provide long-term survival for patients with HCC, a high recurrence rate after surgery is a major problem [3]. Despite progress in molecular biology and cancer therapy in recent years, the overall prognosis for HCC remains dismal, which is mainly attributed to the high incidences of local recurrence, distant metastasis, and therapy resistance [4]. Recently, the tumor microenvironment, which plays an important role in the initiation, progression, recurrence, and metastasis of various tumors, has been intensively studied. Changes of the tumor microenvironment have been closely correlated with cancer-mediated inflammation. Furthermore, an inflammatory response is detectable in tumors that is not causally related to inflammation [5].

High-mobility group box 1 (HMGB1), an evolutionarily conserved, chromatin-binding protein [6, 7], is normally located in the nucleus, where it acts as a DNA chaperon by regulating transcription, replication, recombination, repair, and genome stability [8]. In response to some stimuli, such as hypoxia, it can be released into the extracellular environment [9]. Necrotic cells, particularly those derived from cancer tissues, passively release HMGB1, mediating local inflammation and cancer development [10]. HMGB1 overexpression has been observed in the cells of some cancers such as breast cancer [11], colon cancer [12], gastrointestinal stromal tumors [13], and liver cancer [9]. Through the binding with its receptors, such as RAGE or TLRs, HMGB1 has been associated with tumor-cell survival, progression, and metastasis [14, 15]. Extracellular HMGB1 displays cytokine activity and can promote inflammation by activating macrophages, which represent the main type of inflammatory cells infiltrating tumors [16, 17].

Tumor-associated macrophages (TAMs) are mainly polarized towards an M2 phenotype, which has been associated with angiogenesis, metastasis, and poor prognosis [18]. Accumulating evidence has demonstrated an association between TAM density and unfortunate prognosis in HCC [19–21]. An abundance of CD68 macrophage infiltration in peritumoral liver tissues, but not in tumors, was previously associated with poor prognosis in HCC [22]. Exposure of macrophages to HMGB1 exerts proinflammatory effects by inducing the TLR4-dependent and CD14-dependent release some inflammatory cytokines such as monocyte chemotactic protein 1, interferon gamma-induced protein 10, and macrophage inflammatory protein 1α [23–25].

HMGB1 is associated with clinicopathologic features and has prognostic significance for overall and disease-free survival in patients with HCC after curative hepatectomy [26, 27]. To the best of our knowledge, there are no previous reports of an association between HMGB1 expression and TAM infiltration in tumors and peritumoral tissues in HCC. We therefore aimed to ascertain whether HMGB1 expression is correlated with TAM infiltration in tumors and peritumoral tissues and to determine whether HMGB1 expression correlates with prognosis in patients with HCC.

## Methods

### Patients, specimens, and follow-up

We collected tumoral and peritumoral specimens from 149 patients (82 men and 67 women; mean age: 67.5 years) with pathologically confirmed HCC complicated with cirrhosis who underwent curative liver resection at our institute (Liver Cancer Institute, Zhongshan Hospital, Fudan University). The median tumor size was 4.2 cm (range: 1.5–11.0 cm). Another 36 patients with HBV-negative HCC without liver cirrhosis were also collected to examine the expression of HMGB1 in peritumoral liver tissues. An experienced pathologist examined haematoxylin and eosin-stained sections from each tumor sample to confirm the histological diagnosis and assess the tumor content. Histological diagnoses including the tumor differentiation and encapsulation were made according to the guidelines proposed by the World Health Organization. Clinical profiles and follow-up records after surgical resection were obtained from the medical records of Zhongshan Hospital of Fudan University.

The follow-up procedures were described in our previous reports [28]. Briefly, the patients were monitored by abdominal ultrasonography, serum a-fetoprotein (AFP), and chest radiography with an interval of 2–6 months according to the postoperative time. If recurrence was suspected, computed tomography scanning or magnetic resonance imaging was performed immediately. Treatment modalities after relapse were administered according to a uniform guideline. Overall survival (OS) and recurrence-free survival (RFS) were defined as the interval between surgery and death or disease recurrence, respectively. If recurrence was not diagnosed, the patients were censored on the date of death or last follow-up.

The study was approved by the Qilu Hospital of Shandong University Ethics Committee, and written informed consent for recording and analysis of data was obtained from all patients.

### Immunohistochemical staining and macrophage quantification

HMGB1 expression in paired tumoral and paratumoral liver tissues from the patients was examined by immunohistochemistry. We used CD68 to mark the macrophages in the tissues. Using the semiquantitative scale described previously [29], the HMGB1protein expressions were scored by multiplying the proportion of positive cells and intensity. The proportion of positive cells ranked as follows: 1(≤25 %), 2 (26–50 %), 3 (51–74 %), and 4 (≥75 %). The intensity of nuclear or cytoplasmic staining was evaluated as follows; no staining/background of negative controls (score = 1), weak staining detectable above background (score = 2), moderate staining (score = 3), and intense staining (score = 4). The index was obtained by multiplying the percentage and intensity scores, the score ≤ 8 was defined as low expression, and the score >8 was defined as high expression. Meanwhile, the HMGB1protein expression and CD68+ cells density were also quantified with the method described in our previous study [28]. Under high-power magnification (200×), photographs of two representative fields of each punch were captured by a computerized image system composed of a Leica CCD camera DFC 500 connected to a Leica DM IRE2 microscope and Leica Qwin Plus v3 software (Leica Microsystems Imaging Solution, Cambridge, UK). We measured the area of positive staining in pixels using Image-Pro Plus v6.2 software (Media Cybernetics, Bethesda, MD). The expression of HMGB1protein and the densities of CD68 staining were expressed as the ratio of the positively stained area to the total area of each photograph.

### Statistical analysis

We performed all statistical analyses using SPSS16.0 for Windows (SPSS). Correlations between clinicopathologic characteristics and HMGB1 expression were assessed using the χ^2^ test. We used the Pearsonχ^2^ test or the Fisher exact test to compare qualitative variables and the Student t test or the Spearman correlation test to compare quantitative variables. We used a Kaplan-Meier analysis to determine the survival curves and the log-rank test to compare the survival curves between subgroups. In the survival analyses, we used death from disease and recurrence/metastasis as the endpoints for OS and RFS, respectively. We tested the associations between the variables and survival using univariate and multivariate analyses with the Cox proportional hazard model. The difference between survival curves was analyzed by the log rank test. *p* < 0.05 was considered to be statistically significant.

## Results

### Correlation between HMGB1 expression and clinicopathologic characteristics

**Fig. 1:**
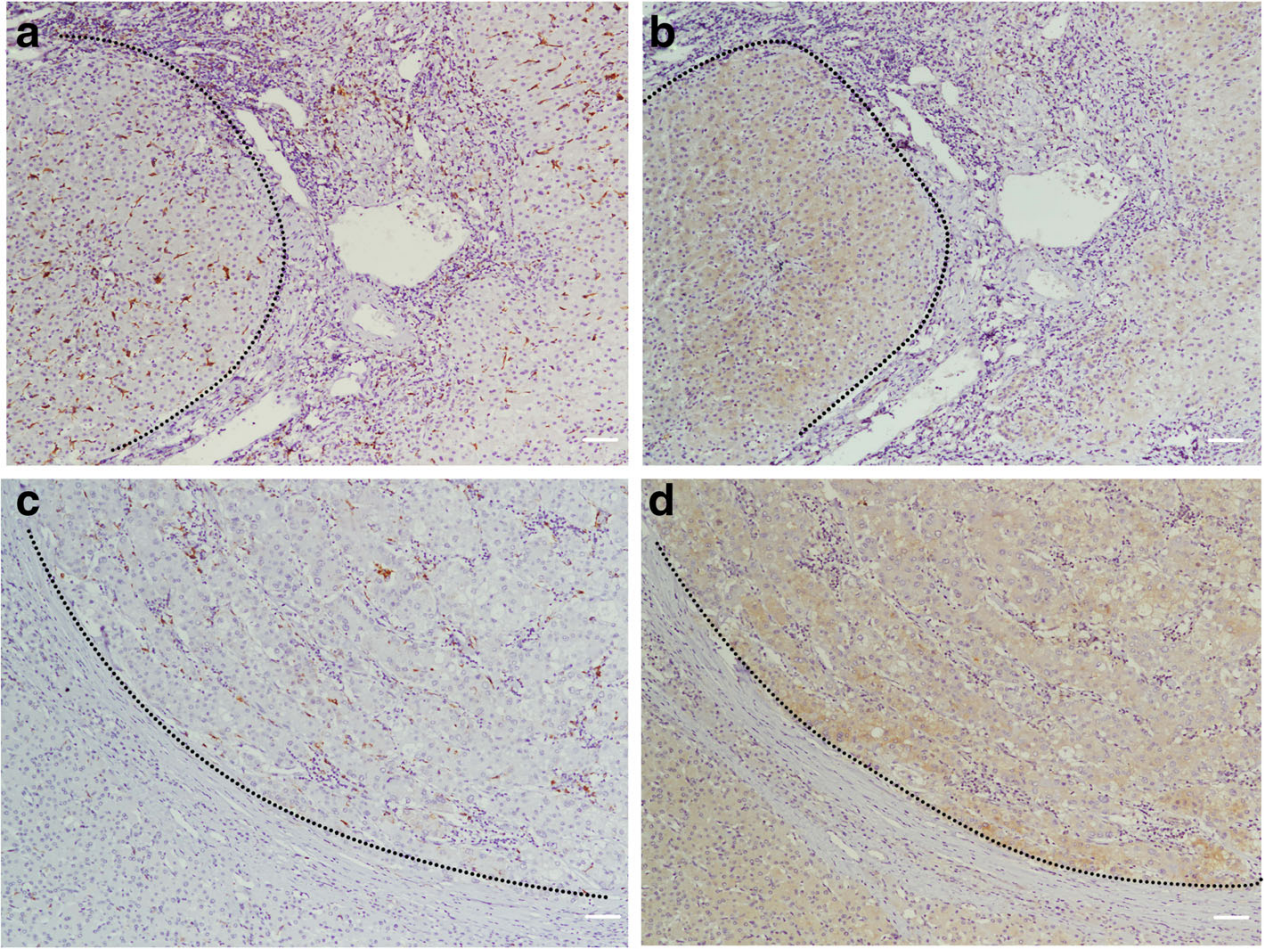
Representative immunostaining for CD68 in peritumoral **a** and tumoral **c** liver tissue and for HMGB1 in peritumoral **b** and tumoral **d** liver tissue. Scale bar, 100 um

**Fig. 2:**
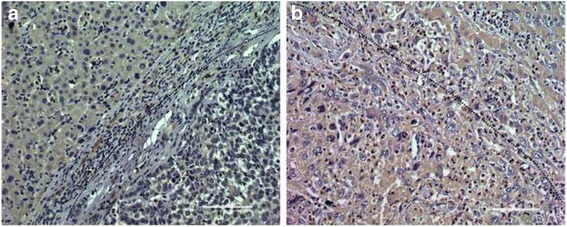
Immunohistochemical staining demonstrated that showed high expression of HMGB1 was associate with high invasiveness of HCC. Representative immunostaining for HMGB1 in tumor with encapsulation (**a**) and without encapsulation (**b**)

HMGB1 immunohistochemical expression showed predominant cytoplasmic staining and little nuclear staining in the tumoral and paratumoral liver tissues (Fig. 1). High HMGB1 expression was detected in 94 (63.1 %) of the tumor tissues and in 59 (39.6 %) of the paired paratumoral tissues. The correlations between clinicopathologic characteristics and HMGB1 expression in the tumoral and paratumoral liver tissues are shown in Table 1. High peritumoral HMGB1 expression was positively correlated with recurrence (*p* = 0.001), tumor encapsulation (*p* = 0.019) (Fig. 2), and advanced BCLC stage (*p* = 0.036). In contrast, intratumoral HMGB1 expression had no statistically significant correlation with tumor encapsulation (Table 2).

**Table 1:** Correlation of HMGB1 expression with known clinicopathologic characteristics in peritumoral liver tissue and in tumor tissue in 149 HCC cases

	Peri-HMGB1 expression			Intra-HMGB1 expression		
Features	Low	High	*p* value	Low	High	*p* value
Age						
≤ 55	55	36		32	59	
> 55	35	23	0.563	23	35	0.277
Gender						
Male	53	29		36	46	
Female	37	30	0.159	19	48	0.055
a-Fetoprotein						
≤ 400 ng/ml	67	43		37	73	
> 400 ng/ml	23	16	0.489	18	21	0.116
Tumor size (mean ± SD, cm)						
≤ 5 cm	75	47		47	75	
> 5 cm	15	12	0.360	8	19	0.262
Encapsulation/none						
Complete	38	36		22	52	
Incomplete	52	23	0.019	33	42	0.051
Tumor differentiation						
I-II	70	45		43	72	
III-IV	20	14	0.491	22	22	0.496
BCLC stage						
I	60	48		35	73	
II-III	30	11	0.036	20	21	0.049
Recurrence						
Yes	27	34		22	39	
No	63	25	0.001	33	55	0.022

**Table 2:** Correlation of CD68^+^ macrophage with known clinicopathologic characteristics in peritumoral liver tissue and in tumor tissue in 149 HCC cases

	Peri-CD68 expression			Intra-CD68 expression		
Features	Low	High	*p* value	Low	High	*p* value
Age, year						
≤ 55	35	56		43	48	
> 55	32	26	0.034	30	28	0.358
Gender						
Male	40	42		39	43	
Female	27	40	0.192	34	33	0.412
a-Fetoprotein (ng/ml)						
≤ 400	46	64		55	55	
> 400	21	18	0.134	18	21	0.411
Tumor size (mean ± SD, cm)						
≤ 5	53	69		61	61	
> 5	14	13	0.280	12	15	0.379
Encapsulation/none						
Complete	38	36		39	35	
Incomplete	29	46	0.082	34	41	0.231
Tumor differentiation						
I-II	51	64		60	55	
III-IV	16	18	0.466	13	21	0.109
BCLC stage						
I	54	54		49	59	
II-III	13	28	0.033	24	17	0.105
Recurrence						
Yes	36	41		37	40	
No	31	41	0.387	36	36	0.471

### Peritumoral HMGB1 expression was upregulated in HBV-positive HCC, but not in HBV-negative HCC

We examined the expression of HMGB1 in peritumoral liver tissues from 36 patients with HBV-negative HCC without liver cirrhosis. Only four of those samples displayed high HMGB1 expression, while the other 32 displayed low HMGB1 expression. Overall, the peritumoral expression of HMGB1 was lower in the patients with HBV-negative HCC than in the patients with HBV-positive HCC (*p* = 0.004), which might be attributed to the background of cirrhosis (Additional file 1: Figure S1).

### HMGB1 expression was associated with peritumoral TAM infiltration

**Fig. 3:**
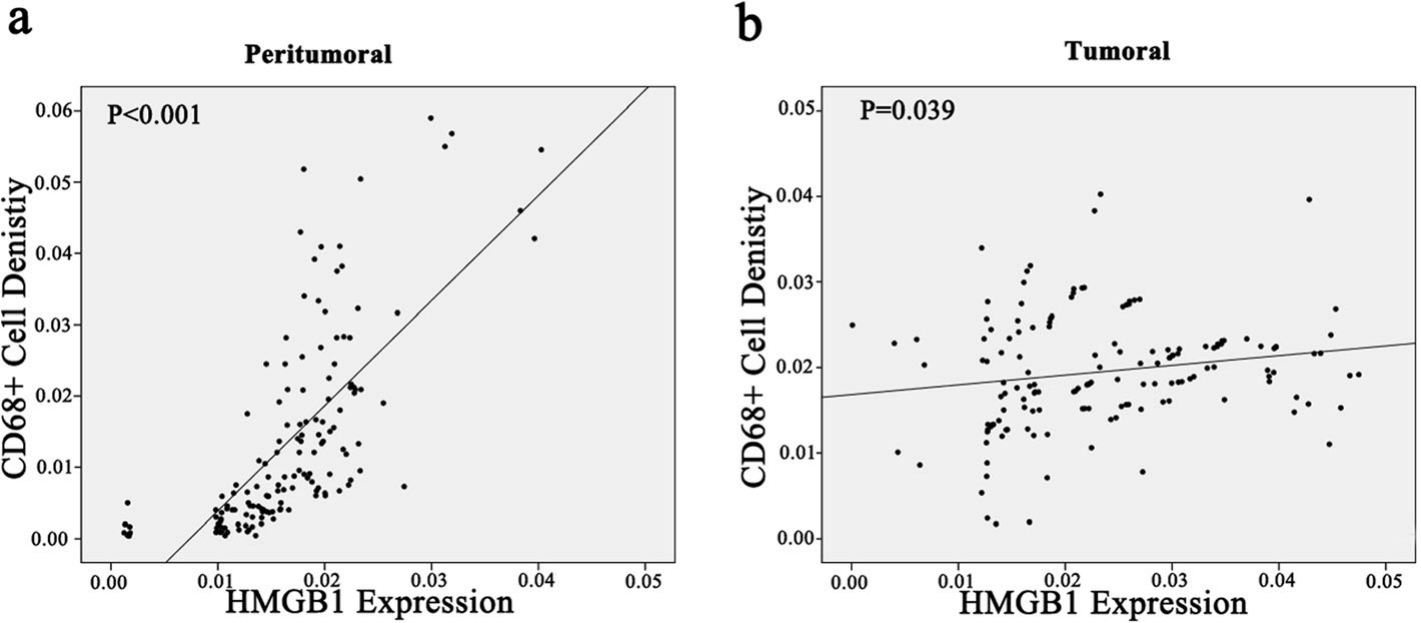
Correlation of HMGB1 expression with CD68^+^ macrophage infiltration in peritumoral and tumoral liver tissues in patients with HCC. Two-demissional plot showed HMGB1 overexpression correlated positively with CD68^+^ macrophage density in peritumoral liver tissue (**a**), while modest correlation was found between HMGB1 expression and CD68^+^ macrophage density in tumor tissues (**b**)

Because HMGB1 could activate human peripheral blood monocytes and induce the production of proinflammatory cytokines, we examined whether macrophage infiltration was associated with HMGB1expression in tumoral or peritumoral liver tissues. As shown in Fig. 3, there was significantly more TAM infiltration in the peritumoral liver tissues with high HMGB1 expression than in the peritumoral liver tissues with low HMGB1 expression. TAM infiltration was high in all of the tumor tissues, however, regardless of the HMGB1 expression level, and there was no significant difference in TAM infiltration in the tumor tissues based on HMGB1 expression. As shown in Fig. 3, HMGB1 overexpression was correlated positively with CD68^+^ macrophage density in the peritumoral liver tissues (*p* < 0.001).

### Prognostic relevance of HMGB1 expression and TAM infiltration in HCC

**Fig. 4:**
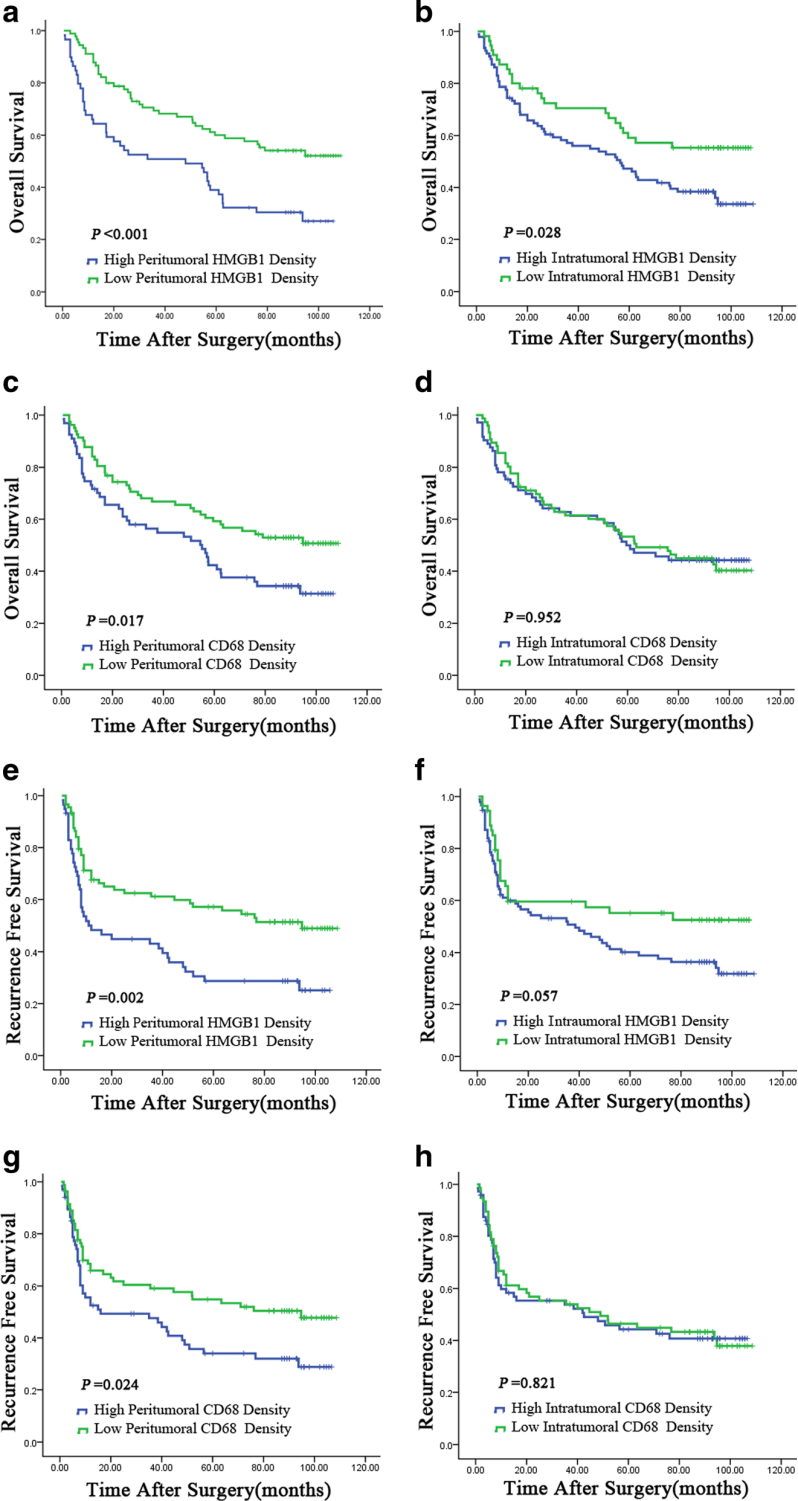
Cumulative overall survival (OS) and recurrence-free survival (RFS) curves of patients with high and low expression of HMGB1 (panels **a**, **b**, **e**, and **f**) and high and low densities of CD68^+^ cell infiltration (panels **c**, **d**, **g**, and **h**) in tumoral and peritumoral liver tissues. In peritumoral liver tissues, the expression of HMGB1 and the density of CD68^+^ cells were both associated with poor OS and RFS (panels **a**, **c**, **e**, and **g**). In tumor tissues, the expression of HMGB1 was associated with poor OS but not RFS (panels **b** and **f**), while the density of CD68^+^ cells could not discriminate patients with different OS or RFS (panels **d** and **h**)

**Fig. 5:**
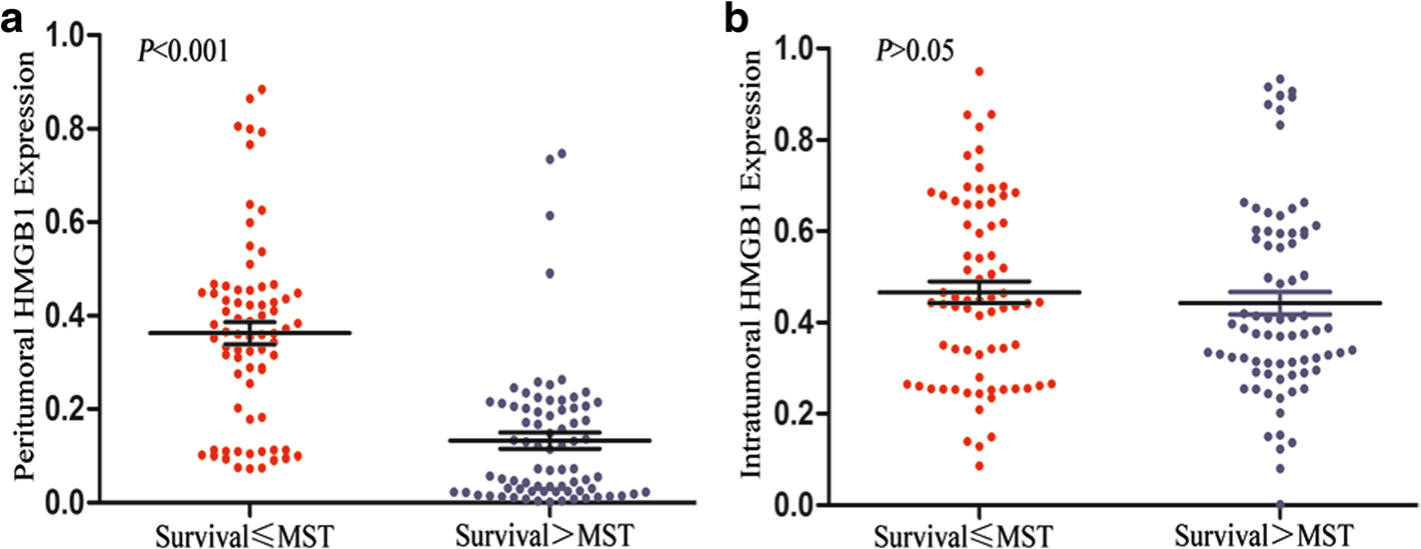
Peritumoral HMGB1 expression in group of bad prognosis (Survival < MST) was significantly higher than those in group of good prognosis (Survival > MST) (**a**), while intratumoral HMGB1 expression in group of bad prognosis was modestly higher than those in group of good prognosis (**b**)

We examined whether the expression of HMGB1 in tumoral and peritumoral liver tissues had value in predicting clinical outcomes or prognosis in HCC. The Kaplan-Meier analysis (log-rank test) showed that HMGB1 overexpression in both the peritumoral liver tissues and the tumor tissues was associated with worse OS (*p* < 0.001 and *p* = 0.028, respectively). Only HMGB1 overexpression in the peritumoral liver tissues was associated with worse RFS, however (*p* = 0.002). The TAM count in the peritumoral liver tissues was significantly associated with both OS and RFS (*p* = 0.017 and *p* = 0.024, respectively), whereas the TAM count in the tumor tissues was associated with neither OS nor RFS (Fig. 4). We also performed the analysis of prognosis and HMGB1. 149 patients were categorized into two groups according to their survival time. Of the two groups, good prognosis,who survived longer than median survival time(MST), were involved in group One, and another group was matched with bad prognosis who survived shorter than MST. We compared the HMGB1 level in two groups. Peritumoral HMGB1 expression in group of bad prognosis was significantly higher than those in group of good prognosis, while intratumoral HMGB1 expression in group of bad prognosis was modestly higher than those in group of good prognosis (Fig. 5).

For further analysis, we investigated whether the HMGB1 expression in the peritumoral or tumoral liver tissues was an independent prognostic variable. We constructed a Cox proportional hazards model using established prognostic factors, HMGB1 expression, and TAM count. The univariate analysis revealed that tumor size, tumor differentiation, BCLC stage, peritumoral and intratumoral HMGB1 expression, and peritumoral TAM count, but not intratumoral TAM count, were independent prognostic factors for OS. In addition, patients with high peritumoral HMGB1 expression had a worse RFS prognosis than those with low peritumoral HMGB1 expression (*p* = 0.002). Univariate analyses also indicated tumor size, tumor differentiation, BCLC stage, peritumoral HMGB1 expression, and peritumoral TAM count to be prognostic factors for RFS.

Multivariate analyses indicated peritumoral HMGB1 expression, peritumoral TAM count, and BCLC stage to be independent prognostic factors for OS. In addition, multivariate analyses indicated peritumoral HMGB1 expression, peritumoral TAM count, and tumor differentiation to be independent prognostic factors for RFS. The multivariate analyses showed no significant association between intratumoral TAM count and either RFS or OS (Table 3).

**Table 3:** Univariate and multivariate analyses of factors associated with survival and recurrence

	OS			RFS		
	P*	Multivariate		P*	Multivariate	
		HR(95 %)	*P*		HR(95 %)	*P*
Age, y	0.855	NA	NA	0.976	NA	NA
Gender, male/female	0.852	NA	NA	0.572	NA	NA
a-Fetoprotein >400 vs. ≤ 200 ng/dL	0.209	NA	NA	0.064	NA	NA
Tumor size, >5 vs. ≤ 5 cm	0.005	1.699(1.203-2.685)	0.038	0.016	1.478(0.856-2.554)	0.161
Encapsulation complete vs. none	0.735	NA	NA	0.942	NA	NA
Tumor differentiation, III–IV vs. I–II	0.026	1.217(0.715-2.072)	0.468	0.015	1.157(1.103-2.584)	0.026
BCLC stage, C vs. B vs. A	0.003	1.783(1.265-2.564)	0.027	0.003	1.768(0.949-3.295)	0.073
Recurrence, yes vs. no	0.062	NA	NA	0.052	NA	NA
Peritumoral HMGB1, high vs. low	0.001	1.872(1.148-3.053)	0.009	0.002	1.697(1.035-2.783)	0.014
Intratumoral HMGB1, high vs. low	0.028	1.270(0.745-2.166)	0.462	0.060	NA	NA
Peritumoral CD68, high vs. low	0.017	1.567(1.058-2.563)	0.039	0.026	1.532(1.127-2.531)	0.037
Intratumoral CD68, high vs. low	0.952	NA	NA	0.823	NA	NA

*Abbreviations*: *y* year, *BCLC* Barcelona clinic liver cancer, *NA* not adopted

*Univariate analysis

## Discussion

Previously, we found that the density of peritumoral CD68^+^ cells was associated with poor RFS and OS [28]. Therefore, we further analyzed the potential roles and prognostic value of HMGB1 and explored the correlation between HMGB1 and macrophage infiltration in patients with HBV-related HCC complicated with liver cirrhosis. High HMGB1 expression in peritumoral liver tissues was correlated with peritumoral macrophage infiltration, was significantly associated with recurrence, incomplete tumor encapsulation, and advanced BCLC stage, and predicted poorer survival for patients with HCC with liver cirrhosis after curative hepatectomy.

HMGB1 is constitutively expressed in the nucleus of tumor cells and can be released by inflammatory cells and by tumor cells [30]. Once released, HMGB1 works as a damage-associated molecular pattern molecule, binding to receptors such as RAGE and TLRs [31]. There is a growing body of evidence that HMGB1 plays pivotal roles in the development and progression of many types of tumors, including HCC [32]. Most of that evidence is based on the direct effects of HMGB1 on tumor cells [33]. The contribution of immune cells in the tumor microenvironment, and particularly the correlation with macrophages, remains elusive. In the immune system, HMGB1 increases the secretion of inflammatory cytokines (IL-1β, IFN-γ, and TNF-α) [34] and acts as a late inflammatory cytokine by activating macrophages in response to LPS [35]. HMGB1 could enhance tumor-cell invasion and promote angiogenesis by supporting the protumoral functions of TAMs by a RAGE-dependent mechanism [36]. Thus, HMGB1 could act as an autocrine/paracrine tumor-growth factor in cancer. A compelling body of evidence has shown the contribution of HMGB1 to the malignant progression of HCC, and high HMGB1 expression predicts a poor prognosis for patients with HCC [27, 37]. To the best of our knowledge, an association between HMGB1 expression and TAM infiltration has not been reported in HCC.

We found that the HMGB1 expression in peritumoral and tumoral liver tissues was correlated with the TAM count and with several conventional clinicopathological parameters. It is therefore worth determining whether HMGB1 expression and TAM count are accurate and reliable prognostic factors in HCC. HMGB1 expression showed predominant cytoplasmic staining and sparse nuclear staining in tumor and paratumoral liver tissues. HMGB1 expression in the peritumoral liver tissues was strongly correlated with higher histological grades and tumor invasiveness, whereas HMGB1 expression in the tumors was only correlated marginally with recurrence and higher BCLC stage. High HMGB1 expression in the peritumoral liver tissues was correlated with peritumoral macrophage infiltration, whereas that in the tumors was not.

Jiang et al. found that HMGB1 overexpression in tumor tissues, rather than that in paratumoral and normal tissues, was correlated with advanced TNM stage, vascular invasion, and capsule invasion [27]. Our results also demonstrated a correlation between HMGB1 overexpression in HCC tumor tissues and poor OS. In contrast to previous results, however, our study also indicated HMGB1 overexpression in paratumoral tissues that was correlated positively with macrophage infiltration and negatively survival. The fact that our study included only patients with HCC complicated with liver cirrhosis might account for the discrepancy with other studies.

HMGB1 acts as a molecular link between hepatocyte death and liver fibrogenesis [38]. Its release has been observed from the hepatocytes of patients suffering from various kinds of liver diseases [39] and was involved in the activation of hepatic stellate cells, which increases extracellular matrix overproduction [40]. Moreover, the serum HMGB1 concentration increased significantly in chronic CCl_4_ intoxication-induced hepatic fibrogenesis [41]. We identified peritumoral HMGB1 overexpression in only 11 % of patients with HBV-negative HCC without liver cirrhosis, probably because of the close association between HMGB1 expression and liver fibrosis, suggesting that HCC and liver fibrosis are both influenced by HMGB1-mediated macrophage stimulation. Further studies are warranted to investigate of the role and regulation of HMGB1 in liver cirrhosis and HCC.

## Conclusions

In summary, we observed high HMGB1 expression levels in peritumoral liver tissues correlated with peritumoral macrophage infiltration. Peritumoral HMGB1 expression had prognostic value in HCC with cirrhosis, suggesting that peritumoral HMGB1 might show promise as new biomarker to predict HCC progression and potential therapeutic targets in HCC and liver fibrosis.

## Additional file

Additional file 1: Figure S1. Immunohistochemical staining demonstrated that peritumoral expression of HMGB1 was higher in the patients with HBV-positive HCC (A, B) than in the patients with HBV-negative HCC (C, D). (TIF 16359 kb)

## Abbreviations

AFP: A-fetoprotein
BCLC: Barcelona-clinic liver cancer
HBV: Hepatitis B virus
HCC: Hepatocellular carcinoma
HMGB1: High-mobility group box 1
MTS: Median survival time
OS: Overall survival
RFS: Recurrence-free survival
TAMs: Tumor-associated macrophages
