# Supplementary figures and images for: High-mobility group protein box1 expression correlates with peritumoral macrophage infiltration and unfavorable prognosis in patients with hepatocellular carcinoma and cirrhosis
Source: BMC Cancer. 2016 Nov 11;16:880. doi: 10.1186/s12885-016-2883-z (PMC5106788; doi:10.1186/s12885-016-2883-z)

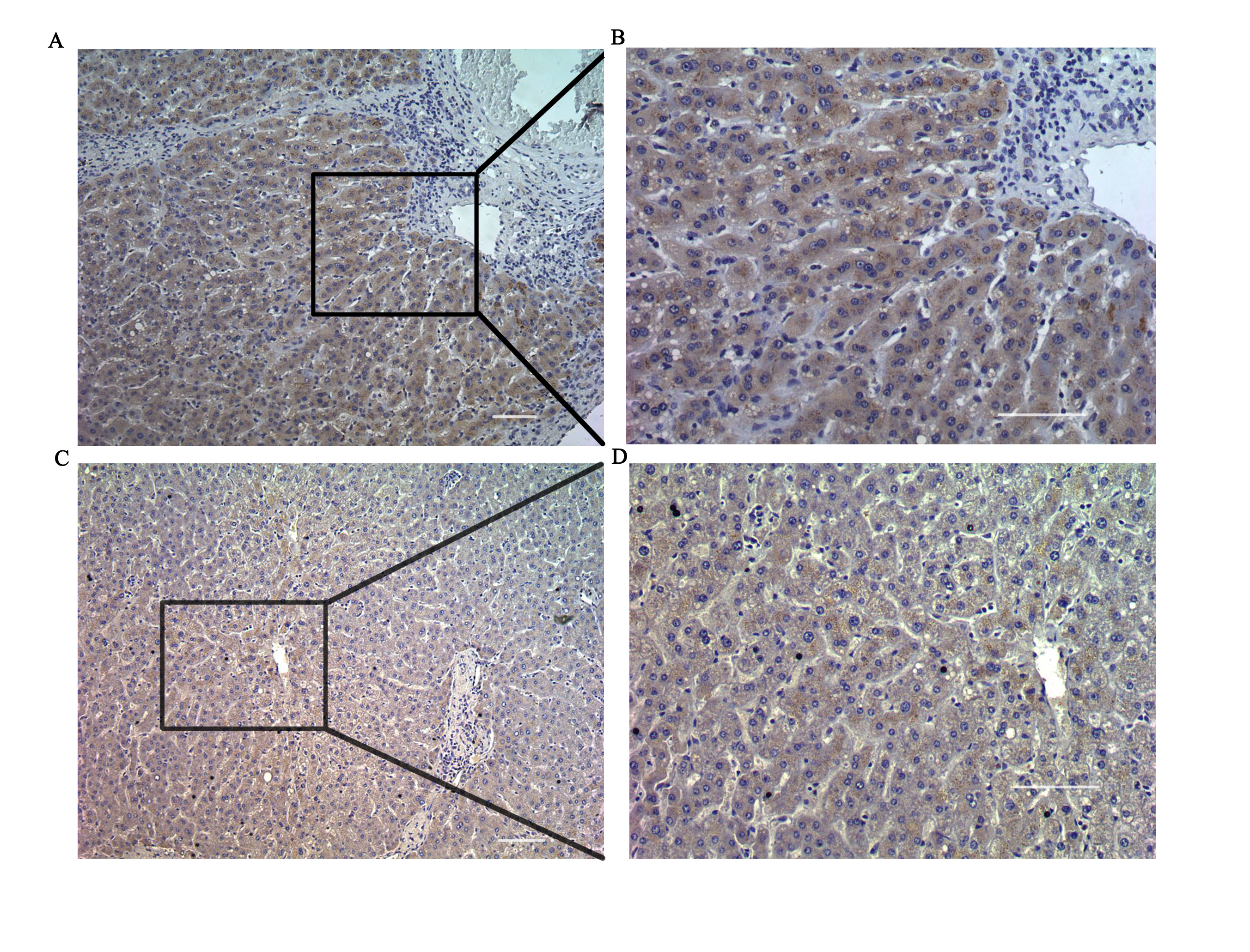

Supplement: Additional file 1: Figure S1. — Immunohistochemical staining demonstrated that peritumoral expression of HMGB1 was higher in the patients with HBV-positive HCC (A, B) than in the patients with HBV-negative HCC (C, D). (TIF 16359 kb) [file 12885_2016_2883_MOESM1_ESM.tif]
